# Photoactivated adenylyl cyclases attenuate sepsis-induced cardiomyopathy by suppressing macrophage-mediated inflammation

**DOI:** 10.3389/fimmu.2022.1008702

**Published:** 2022-10-18

**Authors:** Guofang Xia, Hongyu Shi, Yuanyuan Su, Beibei Han, Chengxing Shen, Shiqiang Gao, Zhong Chen, Congfeng Xu

**Affiliations:** ^1^ Department of Cardiology, Shanghai Sixth People’s Hospital Affiliated to Shanghai Jiao Tong University School of Medicine, Shanghai, China; ^2^ Wusong Central Hospital, Shanghai, China; ^3^ Institute of Physiology, Department of Neurophysiology, Julius-Maximilians-University of Wuerzburg, Wuerzburg, Germany

**Keywords:** macrophage, photoactivated adenylyl cyclase, inflammation, optogenetics, sepsis-induced myocardiopathy, cell-based therapy

## Abstract

Sepsis-induced myocardiopathy, characterized by innate immune cells infiltration and proinflammatory cytokines release, may lead to perfusion failure or even life-threatening cardiogenic shock. Macrophages-mediated inflammation has been shown to contribute to sepsis-induced myocardiopathy. In the current study, we introduced two photoactivated adenylyl cyclases (PACs), *Beggiatoa* sp. PAC (bPAC) and *Beggiatoa* sp. IS2 PAC (biPAC) into macrophages by transfection to detect the effects of light-induced regulation of macrophage pro-inflammatory response and LPS-induced sepsis-induced myocardiopathy. By this method, we uncovered that blue light-induced bPAC or biPAC activation considerably inhibited the production of pro-inflammatory cytokines IL-1 and TNF-α, both at mRNA and protein levels. Further, we assembled a GelMA-Macrophages-LED system, which consists of GelMA—a type of light crosslink hydrogel, gene modulated macrophages and wireless LED device, to allow light to regulate cardiac inflammation *in situ* with murine models of LPS-induced sepsis. Our results showed significant inhibition of leukocytes infiltration, especially macrophages and neutrophils, suppression of pro-inflammatory cytokines release, and alleviation of sepsis-induced cardiac dysfunction. Thus, our study may represent an emerging means to treat sepsis-induced myocardiopathy and other cardiovascular diseases by photo-activated regulating macrophage function.

## Introduction

Sepsis, defined as a life-threatening acute multi-organ dysfunction secondary to infection, manifests the leading cause of death for patients in intensive care units (1). While bacterial infections cause most cases of sepsis, novel coronavirus is believed to be a prevailing cause of sepsis in the current COVID-19 pandemic (2). Under the stimulation of invading pathogens, host immunity may respond excessively and extensively, releasing multiple pro-inflammatory cytokines, which can lead to a deleterious multiorgan failure, referred to as septic shock. Among the patients, about 10% manifested with both hypovolemic and cardiogenic shock, which is a result of cardiomyocyte depression and myocardial dysfunction (3). With sensitive diagnostic techniques for measuring cardiac function evolving, septic cardiomyopathy, a reversible and moderate form of sepsis-induced myocardial injury, was recognized increasingly (4). Both septic cardiogenic shock and septic cardiomyopathy are characterized by innate immune cells infiltration, including macrophages and neutrophils, and proinflammatory cytokines release, such as IL-1β, IL-6, TNF-α, C5a and NO (4–6), these cytokines implicate deleterious effects on cardiomyocyte contractility (7, 8). Obviously, to suppress overactivation of immune cells and cytokine releasing represents one reasonable means of intervention for cardiomyopathy.

Cyclic adenosine monophosphate (cAMP) is a potential second messenger and has pronounced effects on a variety of biological processes, such as development, carcinogenesis, and inflammation. The generation of cAMP is catalyzed by adenylyl cyclase (AC), a superfamily of 10 protein isoforms with disparate localization in different cells (9). cAMP has been shown to perform diverse functions in macrophages, such as inhibition of phagocytosis, microbes killing, and the expression of proinflammatory cytokines (10–13). The work from our team showed that AC6 dampens macrophage-mediated inflammation by endocytosis of TLR4 to the lipid-raft mediated pathway leading to its degradation (12). However, due to the limited specificity of commonly used AC agonists to modulate the concentration of intracellular cAMP, there are limited AC agonists to be used to regulate inflammation and perform translational research (11).

Light-controllable optogenetics technique is a cutting-edge tool to manipulate cellular or subcellular function with negligible invasiveness and high spatiotemporal precision. Light-gated ion channels and neurotransmitter receptors have remarkably promoted the understanding of some intractable neuroscience questions, such as neural circuits, vision restoration, and drug discovery (14–16), and attracted rising attention in other fields recently (17–19). However, there are sporadic studies about the function of light-gated enzymes in regulating inflammation. In the present study, we applied two light-gated cAMP-producing enzymes, bPAC and biPAC, to modulate inflammation in macrophages. Based on the light-induced anti-inflammatory activity of bPAC, we assembled a GelMA-Macrophages-LED device to regulate the cardiac local inflammation in LPS-induced septic myocardiopathy, providing one novel means to regulate inflammation and treat sepsis-induced myocardiopathy with the application of optogenetic techniques *in vivo*.

## Materials and methods

### Animals

Six to eight weeks old C57BL/6J male mice were purchased from Vital River Laboratory Animal Technology (Beijing, CN), and conditioned in-house for 7 days after arrival with commercial diet and tap water available at will. All mice experiments were approved by the Ethics Committee of Shanghai Jiaotong University School of Medicine Affiliated Sixth People’s Hospital.

### LPS-induced sepsis mice model

LPS-induced sepsis model, a recognized mice model to study septic myocardiopathy, was performed as previously described (20, 21). On the day of operation, C57BL/6J mice were injected intraperitoneally with 5 mg/kg *Escherichia coli* O111: B4 LPS (Beyotime Biotech, Shanghai, CN) dissolved in sterile PBS or an equivalent volume of PBS (for control group). The dosage of LPS was chosen based on a preliminary experiment to achieve evident myocardial injury without significant mortality.

### Plasmids construction

The bPAC (*Beggiatoa* sp. photoactive adenylyl cyclase) cDNA (Genbank™ accession number GU461307) modified for expression in mammalian cells was fused with yellow fluorescent protein and inserted into pBK-CMV vector. The biPAC (*Beggiatoa* sp. IS2 photoactive adenylyl cyclase) was synthesized by GeneArt Genesynthesis with codon optimized to mouse according to GeneBank accession No. OQW94152. Then the biPAC cDNA was also fused with yellow fluorescent protein and inserted into pBK-CMV vector.

### Reagents and antibodies

LPS from *Salmonella Minnesota* R595 was acquired from Sigma-Aldrich (St. Louis, MO, US). Anti-GFP antibody (D5.1) and anti-HSP90 antibody (C45G5) was obtained from Cell Signaling Techonology (Danvers, MA, US). Notably, the anti-GFP antibody could also detect YFP-tagged protein exogenously expressed in cells. Anti-CD68 antibody (FA-11) was obtained from Abcam (Cambridge, England). Anti-MPO antibody (AF7494) was purchased from Beyotime Biotech. All other chemicals and reagents were purchased from Sangon Biotech (Shanghai, CN).

### Cell culture and transfection

RAW 264.7 cells were acquired from American Type Culture Collection (ATCC, Bethesda, MD) and cultured as previously described (22). Briefly, RAW 264.7 cells were cultured in DMEM medium supplemented with 10% FBS. Cell viability was determined with trypan blue assay and maintained at a high level all along the experimental procedure. Cells were grown in six-well plates and transfected with pBK-CMV-YFP-bPAC or pBK-CMV-YFP-biPAC, with pBK-CMV vector as control. For transfecting RAW 264.7 cells, TransIT^®^-Jurkat Transfection Reagent (Mirus Bio, Madison, WI) was used as a transfectant according to the manufacturer’s recommendations.

### *In vitro* light activation

RAW 264.7 cells transfected with bPAC or biPAC were handled in red light (Λ = 650 ± 10 nm, AIJIA Electronics, Jiangsu, CN) to avoid uncontrolled activation of bPAC or biPAC. Photoactivation of bPAC or biPAC was achieved by the use of a light-emitting diodes array (blue light, Λ = 460 ± 10 nm, AIJIA Electronics).

### Flow cytometric analysis of culture cells

RAW 264.7 cells were enzymatically dissociated with 0.25% trypsin, harvested, and resuspended into single-cell suspension in cold FACS buffer (PBS with 2% FBS). The cells were then analyzed using FACS Canto Plus (BD Biosciences, Mount View, CA).

### Immunoblotting

Immunoblotting was performed as previously described (23), with slight modification. Briefly, cell lysates were prepared using ice-cold RIPA lysis buffer (Beyotime Biotech) supplemented with protease inhibitor cocktail (Roche, Indianapolis, IN, USA) and phosphatase inhibitor (Roche). Protein samples were separated by electrophoresis with 10% SDS-PAGE gels and transferred to polyvinylidene difluoride membranes. The membranes were probed with indicated primary antibodies. Subsequently, the HRP-conjugated secondary antibodies were incubated for 1 h at room temperature. The immunoreactive bands were detected with West Pico PLUS Chemiluminescent kit (ThermoFisher Scientific, Waltham, MA) using the ECL detection system (Tanon, Shanghai, CN).

### Enzyme-linked immunosorbent assay

Cell culture supernatant, murine plasma or heart lysate were collected and measured using sandwich ELISA kit (Senxiong biotech, Shanghai, CN) according to the instructions of the manufacturer. After adding the stop solution, optical density was measured at 450 nm in a microplate reader Biotek Synergy 2 (Biotek, Winooski, VT, USA). The absolute concentration was calculated based on a standard curve.

### Quantitative polymerase chain reaction (qPCR)

qRT-PCR was performed using the primer pairs as follows: *Il1*, Forward, 5’ - AGCCCATCCTCTGTG -3’ and Reverse, 5’ - TGTGCCGTCTTTCAT -3’; *Tnfa*, Forward, 5’ - CACGCTCTTCTGTCTACTG -3’ and Reverse, 5’ - ACTTGGTGGTTTGCTACG -3’; *Il10*, Forward, 5’ - GCTCTTACTGACTGGCATGAG -3’ and Reverse, 5’ - CGCAGCTCTAGGAGCATGTG -3’; *Arg1*, Forward, 5’ - CTCCAAGCCAAAGTCCTTAGAG -3’ and Reverse, 5’ - AGGAGCTGTCATTAGGGACATC -3’; *Acta2*, Forward, 5’ - GTCCCAGACATCAGGGAGTAA -3’ and Reverse, 5’ - TCGGATACTTCAGCGTCAGGA -3’; *Col1a1*, Forward, 5’ - GCTCCTCTTAGGGGCCACT -3’ and Reverse, 5’ - CCACGTCTCACCATTGGGG -3’; *Tgfb1*, Forward, 5’ - CTCCCGTGGCTTCTAGTGC -3’ and Reverse, 5’ - GCCTTAGTTTGGACAGGATCTG -3’; *18s*, Forward, 5’- ACCGCAGCTAGGAATAATGGA -3’ and Reverse, 5’- CAAATGCTTTCGCTCTGGTC -3’. Total RNA from cells or tissue sample was extracted using TRIZOL Reagent (Sigma Aldrich). Reverse transcription was performed with HiScript II Reverse Transcriptase (Vazyme, Jiangsu, CN) in accordance with the manufacturer’s instructions. Real-time PCR analysis was performed on a Light Cycler 96 Real-Time system (Roche) for 45 cycles, using an SYBR qPCR Master Mix (Vazyme).

### GelMA-macrophage-LED preparation

GelMA hydrogel was purchased from the Engineering For Life (Jiangsu, CN) and prepared according to the instruction. Briefly, GelMA solution was primed by dissolving GelMA in culture medium at a concentration of 10% bearing 0.25% (w/v) lithium phenyl-2,4,6-trimethyl-benzoyl phosphinate (LAP) as the initiator, and then filtering with a 0.22 μm filter for sterility before use. Transfected Raw 264.7 cells were mixed with the GelMA hydrogel, and the cell concentration was adjusted to 5 × 10^6^ cells/ml. The wireless LED (Red: Λ = 620-625 nm, Blue: Λ = 460-470 nm, ADA, Sydney, AUS) was covered by water-proof parafilm (Bemis, Neenah, US), which allow the LED to work well *in vivo*. 20 μl of the GelMA solution containing modified macrophages were pipetted onto water-proofing wireless LED and crosslinked under blue light (3W, 405 nm, EFL, Jiangsu, China) exposure for 1 minute.

### GelMA-macrophage-LED implantation

Mice were anesthetized with 1.5% isoflurane gas using an isoflurane delivery system (RWD Life Science, Shenzhen, CN). After making a small incision (1 cm) on the skin of the left chest, the major and minor pectoral muscles were dissected and retracted. Using a clamp, a small hole was made at the fourth intercostal space to open the pleural membrane. The GelMA-Macrophage-LED device was carefully implanted into the intercostal space and fixed tightly on the costa with a 6-0 silk suture. Finally, the skin was sutured after inflating the lungs to evacuate the air. After implantation, the mice were conditioned in the wireless power generator for wireless light exposure. Sham-operated animals underwent the same procedure without device implantation. 24 hours later, mice were anesthetized with 2% isoflurane, and tissues were subsequently harvested for analyses.

### Histology

Mice were anesthetized with 2.5% isoflurane and intracardially perfused with cold PBS to exclude blood. Heart samples were harvested, fixed by 4% PFA, embedded in paraffin, and cut into 4 μm transverse sections. Hematoxylin-eosin staining were performed on paraffin-embedded section at papillary level to determine the cardiac inflammation and cardiomyocytes necrosis.

### Semiquantitative analysis of histology

The inflammation severity was scored from 0 to 4 (0, no leucocytes infiltrates; 1, small foci of leucocytes between myocytes; 2, large foci of > 100 inflammatory cells; 3, >10% of a cross-section involved; 4. >30% of a cross-section involved) (24). The myocardial damages were scored as score scales for Cardiac Damage Scores (0, Normal histological structure of myofibers; 1, Cardiomyocytes with a few cytoplasmic vacuoles and normal nuclei; 2, Groups of cardiomyocytes with marked cytoplasmatic vacuolization; 3, Cardiomyocytes with hyaline degeneration and karyopicnosis; 4, Cytoplasm fragmentation with karyorrhexis or karyolysis; 5, Tissue necrosis) (25).

### Cell extraction and flow cytometric analysis

Mice were deeply anesthetized with 2.5% isoflurane and intracardially perfused with ice-cold PBS to exclude blood cells. The hearts were dissected, cut into small pieces using fine scissors, and digested in PBS with 1 mg/ml type II collagenase (Worthington Biochemical Corporation, Lakewood, NJ, USA) and 1 mg/ml type IV collagenase (Worthington Biochemical Corporation) for 20 minutes at 37°C with gentle agitation. Subsequently, the hearts were repeatedly aspirated with a pipette, passed through a 70-μm cell strainer, and washed with PBS. The single cells suspensions were incubated with anti-CD16/32 antibody (93, eBioscience) on ice for 15 min. Subsequently, the cells were washed once and incubated with the following antibodies at 4°C for 30 min: anti-CD45-PE (30-F11, eBioscience), anti-CD11b-BV605 (M1/70, BD Bioscience), anti- F4/80-APC (BM8, eBioscience), anti-Ly6G-BV510 (1A8, BioLegend), anti-CD3-BV421 (145-2C, BD Bioscience), anti-CD19-FITC antibodies. The results were displayed as the percentage compared to single cells. Flow cytometric analysis were performed on a FACS Canto plus (BD Biosciences) and analyzed using FlowJo software (Tree Star).

### Immunofluorescent staining

For Immunofluorescent staining, 10 μm frozen heart sections were fixed with 4% PFA for 10 min at room temperature, permeabilized with 1% Triton-X 100 for 30 min at room temperature, and blocked with 5% donkey serum for 1 hour at room temperature. Staining with the indicated primary antibodies in blocking buffer at 4 °C overnight. Samples were then incubated with anti-Rabbit Alexa488 (ThermoFisher Scientific) or anti-Rabbit Alexa594 (ThermoFisher Scientific) for 1 hour at room temperature and then thoroughly washed with PBS 5 times. The samples were stained with DAPI and imaged using OLYMPUS BX53 fluorescence microscopes. Quantification was performed using ImageJ software.

### Echocardiography

Using a Vevo 2100 instrument (VisualSonics, Toronto, ON, Canada) with an MS-400 imaging transducer, we performed transthoracic echocardiography. In detail, 24 hours after modeling, mice were shaved on the chest, anesthetized with 2% isoflurane, and fixed on the echo pad in a supine position. Two-dimensional (2D) images were recorded in the left ventricular long-axis planes, maintaining the heart rate at 500-550 bpm. Left ventricular End-diastolic volume (EDV), end-systolic volume (ESV), stroke volume (SV), and ejection fraction (EF) were captured in M-mode. All measurements were averaged for 3 consecutive cardiac cycles.

### Data and statistical analysis

All data are presented as mean ± SEM. Statistical comparisons were performed using GraphPad Prism v8.3.0 software. The One-way ANOVA or Two-way ANOVA was used for all statistical analyses in this study. Turkey multiple comparisons test was applied for comparing each mean with every other mean (all-pairwise comparisons), and Dunnett multiple comparisons test was used for comparing each mean with the control mean (multiple-to-one comparisons). *p* value less than 0.05 is considered as statistically significant. The numbers of independent replicates (n) are indicated in the figure legends.

## Results

### Overexpression of bPAC and biPAC in RAW 264.7 macrophages

**Figure 1:**
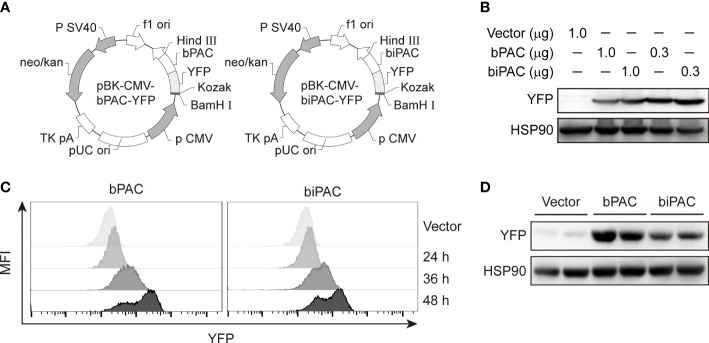
Overexpression of bPAC or biPAC in RAW 264.7 cells. **(A)** Plasmid scheme of pBK-CMV-YFP-bPAC and pBK-CMV-YFP-biPAC. **(B)** Immunoblot of YFP in RAW 264.7 cells transfected with 0.3 μg or 1.0 μg of vector, bPAC or biPAC plasmids and incubated in 37°C for 48 hours. **(C)** Empty Vector, bPAC or biPAC were transfected into RAW 264.7 cells. 24, 36 or 48 hours later, cells were analyzed by flow cytometry. **(D)** 36 hours after transfection, cell lysates from RAW 264,7 macrophages were analyzed by immunoblotting with YFP-specific Abs for expression of bPAC or biPAC.

Photoactive adenylyl cyclase from *Beggiatoa* sp. (bPAC) and *Beggiatoa* sp. IS2 (biPAC) were cloned into pBK-CMV vector to construct plasmid pBK-CMV-YFP-bPAC, and pBK-CMV-YFP-biPAC (**Figure 1A**). Both bPAC and biPAC could be specifically activated by blue light (Λ: 460 ± 10 nm) (17). In this study, we introduced these plasmids into RAW 264.7, a murine macrophage cell line. As RAW 264.7 cells are incalcitrant to transfection, we used transIT-Jurkat transfection reagent to transfect the cells. 2 × 10^6^ cells/well were grown on 6-well plate, and different doses of plasmids (0.3 μg, 1 μg) were used to transfect the cells. After 24 h, 36 h, and 48 h of transfection, we detected the expression of bPAC and biPAC, and found that 0.3 μg plasmids showed a higher transfection efficiency and both bPAC and biPAC expression could be detected from 24 hours post-transfection, with a significant expression after 36 hours (**Figures 1B–D**). So, we thereafter used 0.3 μg and 36 hours post-transfection to perform all other detection and function evaluation.

### Quiescent bPAC and biPAC suppress the transcription of IL-1 and TNF-α

**Figure 2:**
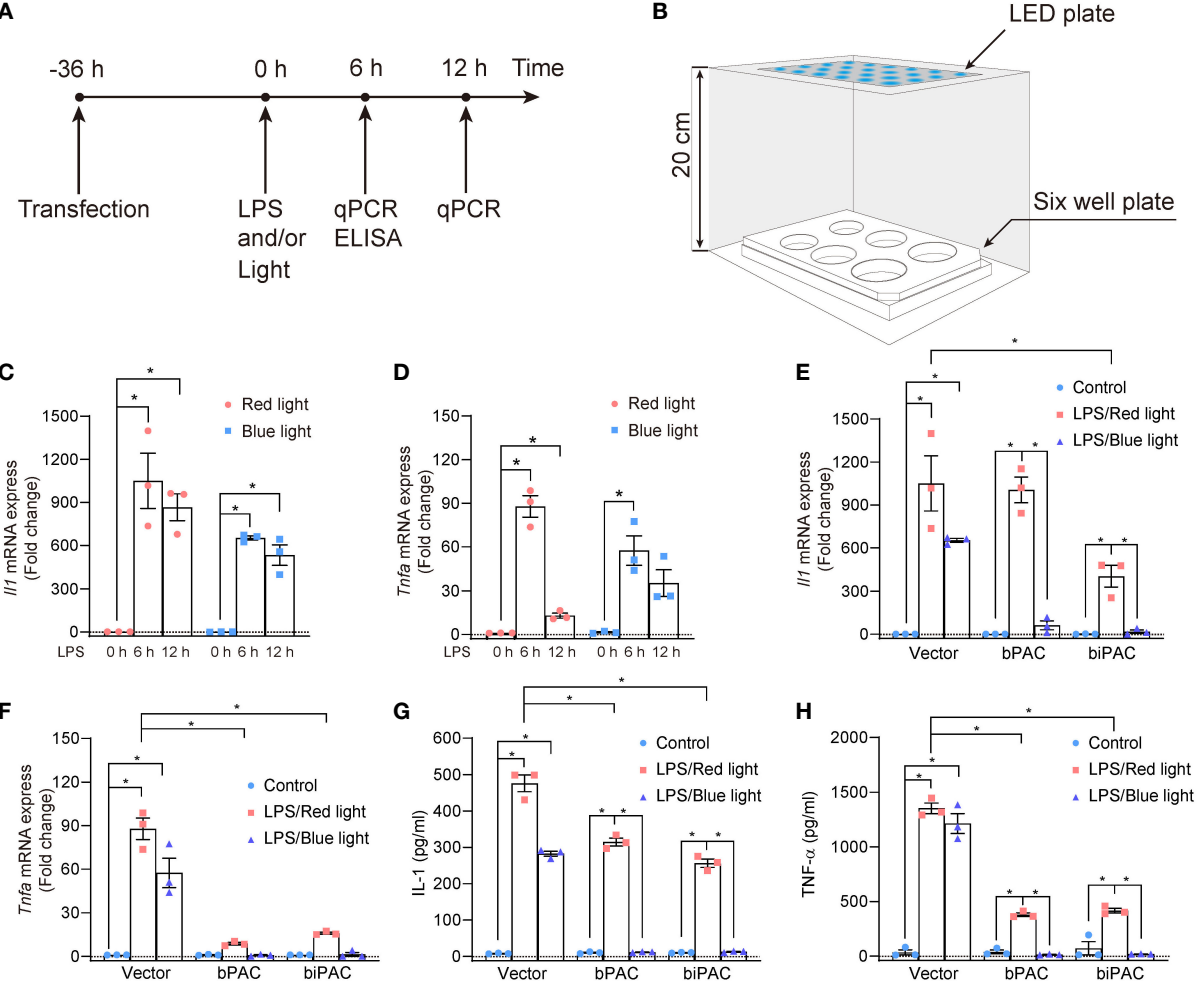
Inflammation inhibition by bPAC and biPAC expression. **(A)** Schematic treatment of RAW 264.7 cells; **(B)** Schematic sketch shows the illumination device used to manipulate the RAW 264.7 cells. The whole facility was covered by tin foil paper, and placed in thermostatic cell incubator. **(C, D)** RAW 264.7 cells were transfected with vector and incubated for 36 hours. After that, Cells were treated with LPS (100 ng/ml) for 6 or 12 hours under light exposure, then lysed, and analyzed by qPCR. RAW 264.7 cells were transfected with vector, bPAC or biPAC plasmids and incubated in 37°C for 36 hours. Then, cells were treated with 100 ng/ml LPS or PBS under red light or blue light. **(E, F)** 6 hours later, cells were harvested for qPCR analysis of *Il-1* or *Tnfa*. **(G, H)** 6 hours later, cell culture supernatant was harvested for ELISA of IL-1 or TNF-α. Data are mean ± SEM of three independent experiments. *p<0.05.

Lipopolysaccharide (LPS), as a ligand of Toll-like receptor 4, activates the downstream pathways (NF-κB and IRF3) in diverse immune cells, leading to the production of a series of proinflammatory cytokines, such as interleukin-1 (IL-1), tumor necrosis factor-a (TNF-α). Adenylyl cyclase (AC) 6 has been shown to dampen inflammation induced by LPS stimulation in macrophages by lipid-raft mediated endocytosis of TLR4 which leads to its degradation (12). In order to evaluate whether bPAC and biPAC induction could regulate inflammation in macrophages, we established the platform of illumination for plates (**Figures 2A, B**). Indeed, LPS stimulates the production of proinflammatory cytokines, such as IL-1, and TNF-α, and reach the peak at about 6 hours post-stimulation (**Figures 2C, D**). With the help of dark control, we found that either red light or blue light showed no influence on the trends of cytokine production in RAW 264.7 cells (data not shown). Therefore, the transcription level after 6 hours of LPS stimulation was used as a readout for the following experiments. In addition, we found that bPAC and biPAC under red light reduced the transcriptions of pro-inflammatory cytokines, especially the transcription of Tnfa, which might due to feeble ambient light interference (**Figures 2E–H**).

### Light-control inhibition of macrophages proinflammatory function

To further investigate the light-induced inhibition of inflammation by bPAC and biPAC, we used blue light/red light to illuminate the RAW 264.7 cells transfected with bPAC and biPAC for 6 hours. After illumination, the RAW 264.7 cells were collected, and total RNA was extracted to evaluate the expression of cytokines by qPCR, while the supernatant was also collected for detecting production of cytokines by ELISA. Our data demonstrated that blue light illumination displayed considerable inhibition of inflammation in RAW 264.7 cells if bPAC and biPAC were activated, based on the expression and production of IL-1 and TNF-α (**Figures 2E–H**). All considered, our data showed that both bPAC and biPAC contribute to the inhibition of inflammation induced by blue light, making optogenetics a possible tool to regulate inflammation.

### Feasibility and safety of GelMA-macrophage-LED device implantation

**Figure 3:**
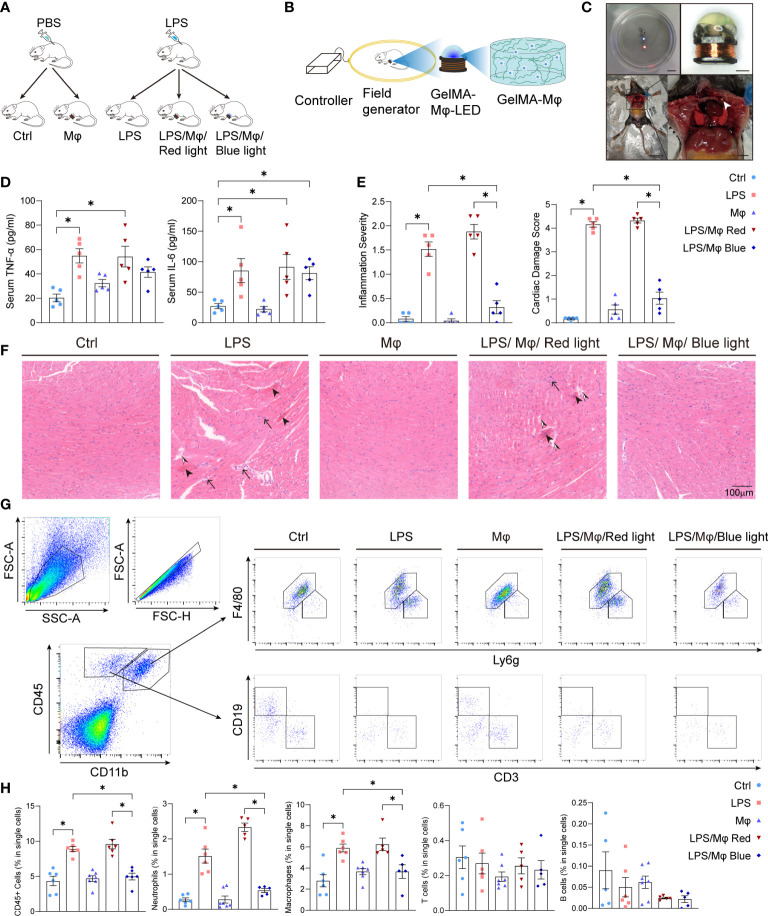
Light-controlled therapeutic effect of modified macrophages on sepsis-induced myocardiopathy. **(A)** Diagram of the study procedure showing LPS administration and GelMA-Macrophage-LED device implantation in C57BL/6 mice. **(B)** Diagram of the wireless LED illumination system and GelMA-Macrophage-LED device. **(C)** Photographs showing wireless LED-controlled modified macrophages and implantation. Upper left: Watertightness treatment made the wireless LED work well in normal saline solution. Scale bar: 1 cm. Upper right: photograph of the wireless LED attached with photo-crosslinking hydrogel. Scale bar: 1 mm. Below: implantation of GelMA-Macrophage-LED into the intercostal space of mice. Left scale bar: 3 cm. Right scale bar: 5 mm. **(D)** IL-6 and TNF-α in sera of mice in control group, LPS group, Mφ group, LPS/Mφ/Red light group, and LPS/Mφ/Blue light group were determined by ELISA. Data are shown as mean ± SEM (n = 5 for each group). **(E)** Semiquantitative analysis of histology of mice in the indicated groups. Data are shown as mean ± SEM (n = 5 for each group). **(F)** Representative figures of heart histology in murine heart from the indicated groups by H&E staining. Sepsis-induced myocardiopathy was characterized by contraction bands necrosis (arrow head), leukocytes infiltration (black arrow) and interstitial edema (black and white arrow head). **(G)** Gating strategy for Cardiac immune cells. The representative flow cytometric plots of macrophages, neutrophils, T cells and B cells in each group. **(H)** The percentage of CD45^+^ cells, macrophages, neutrophils, T cells and B cells relative to total cell population in each group. Data are shown as mean ± SEM (n = 5 mice per group). *p<0.05.

Cell therapy trials have shown that macrophage implantation evidently rejuvenated the infarcted area and improved cardiac function (26). To validate the feasibility of the bPAC-modified macrophages therapeutic scenario *in vivo*, we established sepsis-induced myocardiopathy mice model (**Figures 3A–D**). Twenty-four hours after LPS injection, mice presented characteristic symptoms of sepsis: diarrhea, malaise and piloerection, and elevated plasma TNF-α and IL-6 levels (**Figure 3D**). Then, we constructed the GelMA-macrophage-LED device and implanted it into the intercostal space in healthy mice (**Figures 3B, C**). GelMA-macrophage-LED device implantation did not induce evident cardiac injury nor any death in treated mice (**Figure 3F**). We observed no visible effect on systemic inflammation by light-activated modified macrophage implantation (**Figure 3D**).

### Optogenetic modified macrophages ameliorate the inflammatory response in sepsis-induced cardiomyopathy

**Figure 4:**
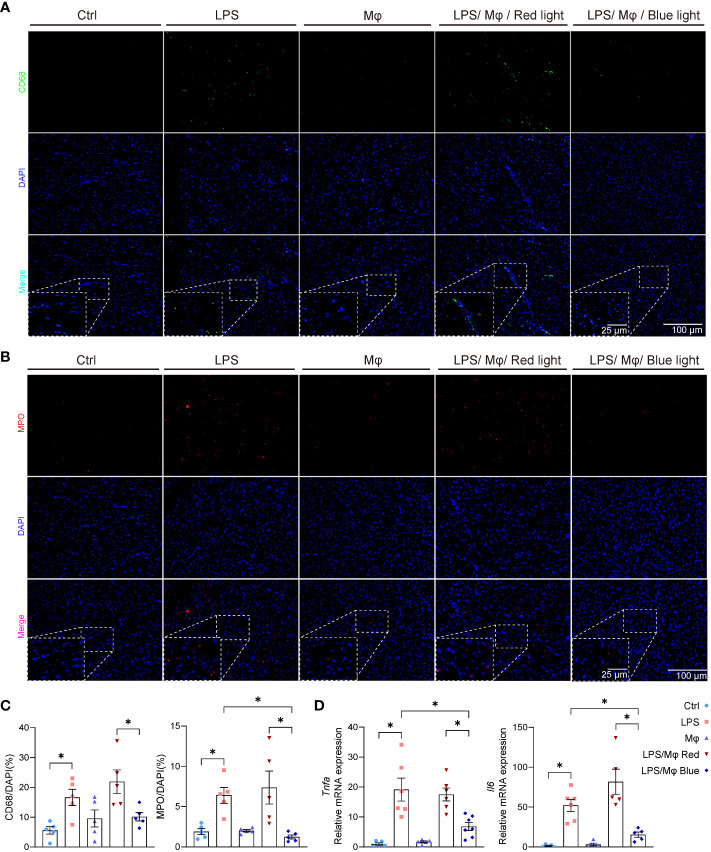
Light-activated inhibition of leukocytes infiltration and pro-inflammatory cytokines release in septic heart. **(A)** Representative immunofluorescence staining of CD68 and nuclear (DAPI) staining in murine heart from control group, LPS group, Mφ group, LPS/Mφ/Red light group, and LPS/Mφ/Blue light group mice at day 1 after LPS injection. **(B)** Representative immunofluorescence staining of MPO and nuclear (DAPI) staining in murine heart from mice in the indicated groups at day 1 after LPS injection. **(C)** Quantification of the immunofluorescence staining of CD68 or MPO. The results are shown as the percentage of CD68-positive cell number or MPO-positive cell number to DAPI-positive cell number. Data are shown as mean ± SEM (n = 5 mice per group). **(D)** qPCR quantification of *Tnfa* and *Il6* transcription level in heart tissue from mice in the indicated groups at day 1 after LPS injection. Data are shown as mean ± SEM (n = 5 mice per group). *p<0.05.

To validate the impact of the modified macrophages on local myocardium, we harvested the heart tissue and examined the cardiac local inflammation 24 hours after the LPS injection and device implantation. Under blue light activation, modified macrophages significantly prevented the infiltration of leukocytes into the myocardium, interstitial edema and cardiomyocytes necrosis (**Figure 3F**). Next, we attempted to characterize the leukocytes that infiltrated into myocardium and caused myocardial injury. Flow cytometric analysis revealed that macrophages and neutrophils are the dominating immune cells infiltrated into myocardium and are specifically regulated by light-controllable modified macrophages (**Figures 3G, H**). Immunofluorescent staining of CD68 and myeloperoxidase (MPO) also demonstrated pronounced inhibition of macrophages and neutrophils infiltration by blue light (**Figures 4A–C**). Consistent with the suppression of leukocytes infiltration, pro-inflammatory cytokines production such as *Tnfa* and *Il6* was substantially inhibited by light-activated modified macrophages (**Figure 4D**), while the anti-inflammatory cytokines, such as *Il10* and *Arg1*, and pro-fibrotic mediators, such as *Tgfb1 and Acta1*, were considerably enhanced (**Supplementary Figures 1B, C**). In addition, we also observed significant increase for the mRNA transcription of *Il10*, *Arg1* and *Tgfb1* in blue light radiated RAW264.7 cells with bPAC-expression (**Supplementary Figure 1A**). These results accentuate that modified macrophages regulated the inflammation in the heart through multiple pathways.

### Optogenetic modified macrophages improve the cardiac function after LPS treatment

**Figure 5:**
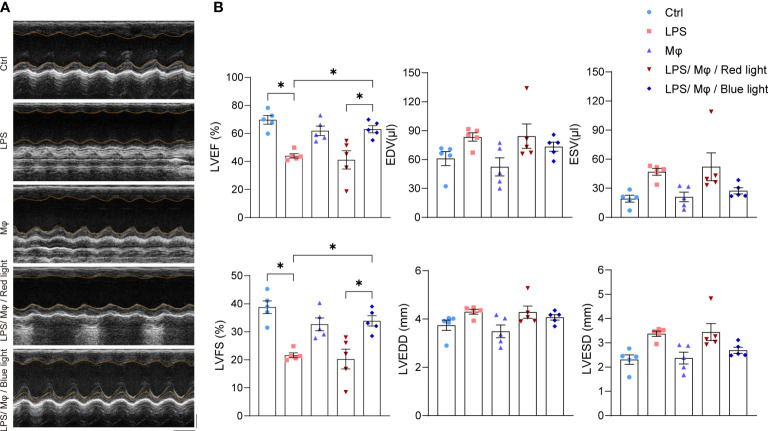
Modified macrophages ameliorate cardiac function in septic heart. **(A)** Representative echocardiographic images of mice in control group, LPS group, Mφ group, LPS/Mφ/Red light group, and LPS/Mφ/Blue light group. **(B)** Quantitative analysis of left ventricular end-diastolic diameter (LVEDD), LV end-systolic diameter (LVESD), LV end-diastolic volume (LVEDV), LV end-systolic volume (LVESV), LV ejection fraction (EF), LV fractional shortening (FS) of mice in the indicated groups. Data are shown as mean ± SEM (n = 5 mice per group). *p<0.05.

Cardiac dysfunction comprises a large component of sepsis-induced multiorgan failure (20). In our animal model, we found that 5 mg/kg LPS was sufficient to impact left ventricular systolic function (**Figure 5**). To determine whether the GelMA-macrophage-LED device could rescue cardiac function under blue light control, we performed echocardiography 24 hours after LPS challenge. The blue light exposure resulted in a significant improvement in ejection fraction (63.10 ± 2.562% in LPS/Mφ/Blue light group, 41.13 ± 6.498% in LPS/Mφ/Red light group) and fractional shortening (33.90 ± 1.839% versus 20.28 ± 3.519%, respectively). Thus, the deterioration of left ventricular systolic function was ameliorated under blue light activation in mice implanted with GelMA-macrophage-LED device (**Figure 5**). Taking together, our work reveals that modified macrophages with blue light-activated bPAC show potent effect to inhibit cardiac inflammation in LPS-induced septic myocardiopathy.

## Discussion

There are more than ten AC isoforms with different tissue distribution and subcellular compartmentalization, which are subsequently involved in different biological processes. However, there are no specific pharmacological agonist for most of them, or with low efficiency, which greatly limited the application of AC isoform-specific regulation. In this study, instead of pharmaceutical agonists, photoactive adenylyl cyclases, bPAC or biPAC were introduced into macrophages to investigate the modulation of inflammation. Our results showed that the light-induced activation of either bPAC or biPAC inhibit inflammation with high efficiency and precision. Combining the bPAC transfected macrophages, photo-crosslinkable hydrogels and wireless LED, we provide one novel means to regulate inflammation and to treat heart diseases with the application of optogenetic techniques.

Both bPAC and biPAC are from chemolithoautotrophic bacteria *Beggiatoa*, and contain catalytic domains homologue to class III adenylyl cyclases (27), which consistently exist in prokaryotes and eukaryotes. The biPAC showed about 3.5 times lower dark activity than bPAC and a higher L/D ratio (ratio of light activity to dark activity) of ~2300 (28). In our study, both bPAC and biPAC showed somewhat inhibition against proinflammatory cytokines production under red light. Probably this was caused by the dark activity of bPAC and biPAC, which depends on the expression level and endogenous cAMP phosphodiesterase activity of the RAW 264.7 cells. While bPAC or biPAC totally limited the transcription of pro-inflammatory cytokines under blue light, which could specifically activate bPAC or biPAC. In this study, we also observed that bPAC or biPAC activation facilitated the elevation of anti-inflammatory cytokines, and even for some pro-fibrotic cytokines, suggesting the complicated pathways mediated by cAMP production on inflammation. These effects make bPAC or biPAC activation a potential tool to dampen excessive inflammation.

Interestingly, in quiescent condition, bPAC showed stronger inhibition activity against IL-1 production, while biPAC demonstrated more vigorous against TNF-α transcription (**Figures 2E, F**). There is a consensus that the transcription of both IL-1 and TNF-α are mediated by NF-κB [15]. But it seems that bPAC or biPAC may modulate the expression of IL-1 and TNF-α through diverse signaling pathways, which is certainly deserved to be further explored. Furthermore, the light-activated cAMP phosphodiesterases (29, 30) can be applied together with PACs for bidirectional regulation of immune cells. Actually, we are working on constructing subcellular optogenetics molecules through anchor proteins such as AKAP79, as the compartment of AC expression also has substantial consequence on its biological function.

In this study, we still go further by introducing an innovative cell-based therapy to treat sepsis-induced myocardiopathy. The management of sepsis-induced myocardiopathy includes three approaches: infection control, heamodynamic stabilization and immunomodulatory therapies, which is the objective of prevailing research. The GelMA-Macrophages-LED device implantation showed negligible surgical injury, rare mortality and good biological compatibility in murine model. Under red light, GelMA-Macrophages-LED device did not exert its anti-inflammatory effect, while blue light reduced leukocytes infiltration and inhibited pro-inflammatory cytokines production, alleviating cardiomyocyte death and heart failure. Thus, using GelMA-macrophage-LED device, we precisely controlled the cardiac inflammation in LPS-induced sepsis and protected heart from inflammation-induced necrosis.

The difficulty for the treatment of sepsis-induced myocardiopathy is the contradiction between efficient antibacterial therapy, heamodynamic stabilization and cardioprotection. Norepinephrine with dobutamine, the substantial inotropic and vasoactive agent, is associated with cardiomyocytes damage and portends poor prognosis in septic patients (31). Systemic anti-inflammatory therapy, including targeted therapy and glucocorticoid administration, could not balance the control of excessive inflammation and clearance of invading pathogens mediated by inflammatory response (32). We applied the GelMA-macrophage-LED device to control cardiac inflammation and improve cardiac function, without affecting antimicrobial function of systemic inflammation and damaging cardiomyocyte.

All together, in our study, we employ light-crosslink GelMA to load the macrophages transfected with photoactive adenylyl cyclase, leading to reliably delivery of the macrophages in contact with the myocardium. Upon blue light activation, macrophage-mediated inflammation induced by LPS could be largely suppressed, providing a potential cell therapy for diverse cardiovascular diseases. With the application of appropriately modified cells and bio-compatible wireless micro-LED, it is promising to achieve precise spatial and temporal regulation of more inflammation-related diseases and application in clinical trials.

## Data availability statement

The raw data supporting the conclusions of this article will be made available by the authors, without undue reservation.

## Ethics statement

The animal study was reviewed and approved by the Ethics Committee of Shanghai Jiaotong University School of Medicine Affiliated Sixth People’s Hospital.

## Author contributions

CX, ZC, GX, and HS conceived and designed the study. CX, ZC, HS, GX, YS, and BH analyzed the data and wrote the manuscript. GX, HS, and YS performed the experiments and acquired the data. CX, ZC, SG, and CS critically viewed and supervised the study and revised the article. All authors read and approved the final manuscript.

## Funding

This work was supported by National Nature Science Foundation of China 31870885, 82171812 (to CX) and Shanghai Bureau of Public Health 201840173 (to CX). The funders play no role in study design, data collection, data analysis, interpretation, and writing of the report.

## Conflict of interest

The authors declare that the research was conducted in the absence of any commercial or financial relationships that could be construed as a potential conflict of interest.

## Publisher’s note

All claims expressed in this article are solely those of the authors and do not necessarily represent those of their affiliated organizations, or those of the publisher, the editors and the reviewers. Any product that may be evaluated in this article, or claim that may be made by its manufacturer, is not guaranteed or endorsed by the publisher.
