# Supplementary material for: Photoactivated adenylyl cyclases attenuate sepsis-induced cardiomyopathy by suppressing macrophage-mediated inflammation
Source: Front Immunol. 2022 Oct 18;13:1008702. doi: 10.3389/fimmu.2022.1008702 (PMC9624221; doi:10.3389/fimmu.2022.1008702)
Supplement: Supplementary file 1 [file DataSheet_1.pdf]

Supplementary Material

Photoactivated adenylyl cyclases attenuate sepsis-induced cardiomyopathy by suppressing macrophage-mediated inflammation

Guofang Xia[†], Hongyu Shi[†], Yuanyuan Su, Beibei Han, Chengxing Shen, Shiqiang Gao, Zhong Chen*, Congfeng Xu*

***Correspondence:**

Congfeng Xu (cxu@shsmu.edu.cn) or,

Zhong Chen (zhongchen7498@hotmail.com)

Supplementary Material

Supplementary Figure

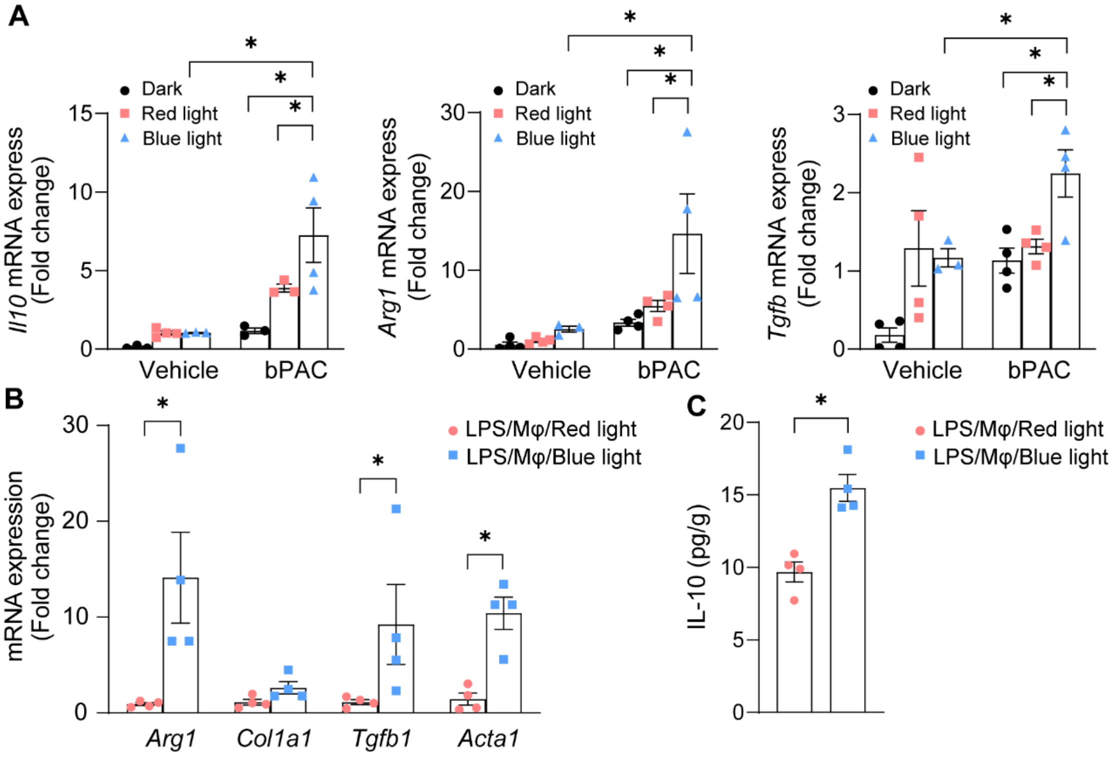

Supplementary Figure 1. The anti-inflammatory and pro-fibrotic cytokines production in bPAC-transfected macrophages and heart.

A. 2×10^6 RAW264.7 cells were cultured in 6-well plate overnight and transfected with 0.3 μg bPAC plasmid. 36 hours later, the cells were treated with 100 ng/ml LPS and cultured in dark or under light exposure for 6 hours, and the RNA was extracted using TRIZOL reagent for RT-qPCR analysis. **B and C**. C57BL/6j mice were implanted with GelMA-Macrophage-LED, injected intraperitoneally with 5 mg/kg LPS and conditioned in wireless power generator for 24 hours. 24 hours later, mice were sacrificed and heart RNA was extracted for RT-qPCR analysis (**B**); and 20 mg heart sample was lysed with 200 μl PBS to measure the IL-10 with ELISA assay (**C**). Data are shown as mean ± SEM of at least three independent experiments or samples from 4 mice. $*p < 0.05$.
